# The 90% effective dose (ED_90_) of esketamine for inhibiting responses to intraoperative motor stimulation during ambulatory hysteroscopy: a biased-coin up-and-down sequential allocation trial

**DOI:** 10.1186/s12871-026-03687-1

**Published:** 2026-02-14

**Authors:** Ruiqi Zhang, Chen Wang, Lin Wang, Wenyue Kang, Zhihua Wang

**Affiliations:** 1 Department of Anesthesiology, Hainan General Hospital (Hainan Affiliated Hospital of Hainan Medical University), Haikou, 570311 China; 2 Department of Anesthesiology, Linfen Central Hospital, Linfen, 041000 China

**Keywords:** Esketamine, Remimazolam, Opioid-free anesthesia, Ambulatory hysteroscopy, Effective dose 90

## Abstract

**Background:**

Esketamine and remimazolam co-administration presents a viable opioid-free anesthetic alternative for outpatient hysteroscopy. This study was designed to ascertain the ED_90_ of esketamine for inhibiting intraoperative movement responses in patients undergoing ambulatory hysteroscopy.

**Methods:**

From February 2024 to May 2024, 50 patients undergoing ambulatory hysteroscopy were enrolled. An anesthetic regimen combining esketamine and remimazolam with 50 mg flurbiprofen axetil was employed. The initial esketamine dose was 0.3 mg/kg, with subsequent doses determined using a biased-coin design (9:1). The primary endpoint was the ED_90_ of esketamine for inhibiting movement responses. Secondary outcomes included anesthesia recovery time, Visual Analog Scale (VAS) scores, and adverse events.

**Results:**

The ED_90_ of esketamine was 0.405 mg/kg (95% CI: 0.370–0.494). Mean anesthesia recovery time was 12.1 ± 2.0 min. No respiratory depression, hypertension, hypotension, or bradycardia occurred. Mild dizziness was observed in 38% of patients; other adverse events (postoperative nausea and vomiting 4%, coughing 4%, hiccups 4%, transient diplopia 2%) resolved spontaneously. Postoperative VAS scores ranged from 0 to 4 at PACU admission (4% reporting 4), decreasing to 0–1 within four hours.

**Conclusions:**

In ambulatory hysteroscopy under remimazolam-based anesthesia with preemptive analgesia using flurbiprofen axetil, the ED_90_ of esketamine required to suppress intraoperative body movement was 0.405 mg/kg (95% CI: 0.370–0.494).

**Supplementary Information:**

The online version contains supplementary material available at 10.1186/s12871-026-03687-1.

## Introduction

Ambulatory hysteroscopy is currently recognized as a preferred approach for the diagnosis and treatment of cervical and uterine cavity disorders, given its minimally invasive nature, rapid recovery, and high diagnostic accuracy. However, procedural pain remains clinically relevant; 35.5% (1829/5151) of women reported high procedural pain scores (70–100 mm on a 100-mm VAS) in a national benchmarking outpatient hysteroscopy survey [1]. In addition, up to 30% of patients may have autonomic responses such as pallor, nausea, and fluctuations in blood pressure and heart rate [2]. These reactions can significantly affect patient comfort and outcomes, requiring anesthetic interventions [3, 4]. Traditionally, propofol combined with opioids is used for anesthesia; however, this combination is often linked to side effects, including pain at the injection site, suppression of respiratory and cardiovascular functions, and opioid-induced issues like nausea and respiratory depression [5].

Opioid-free anesthesia (OFA) has been recognized as a viable alternative, focused on reducing opioid-associated risks while maintaining effective sedation and analgesia [6]. OFA is particularly beneficial for outpatient procedures due to its ability to preserve respiratory and cardiovascular stability and promote faster recovery [7–9]. Studies show that OFA improves pain management, reduces nausea and vomiting, and lowers overall opioid consumption, enhancing recovery outcomes [10–12].

Esketamine, a non-competitive N-methyl-D-aspartate (NMDA) receptor antagonist, holds potential as a critical component in OFA. It provides potent sedation and analgesia with a rapid onset, greater efficacy, and fewer side effects than ketamine [13]. Remimazolam, an ultra-short-acting benzodiazepine, complements esketamine by mitigating psychiatric side effects such as dissociation and nightmares while offering a favorable safety profile with minimal respiratory and cardiovascular depression [14]. Preventive analgesia with agents like flurbiprofen axetil further enhances postoperative comfort and recovery [15].

Previous research has demonstrated that the co-administration of esketamine and remimazolam is efficacious for short procedures such as painless gastroscopy and minor surgeries, with good tolerability. However, the optimal dose of esketamine in combination with remimazolam and flurbiprofen axetil for effectively suppressing intraoperative motor responses during ambulatory hysteroscopy remains unclear. This study utilizes a Biased-Coin Up-and-Down Sequential Allocation Trial to determine the ED_90_ of esketamine, with the goal of optimizing anesthetic protocols and enhancing patient outcomes in ambulatory hysteroscopy.

## Methods

### Study design and ethics

Registered on February 2, 2024, at www.chictr.org.cn (ChiCTR2400080615), this study was granted ethical approval by the Institutional Review Board of Hainan General Hospital (Approval No. YL-2023-198). Written consent was obtained from all participants.

### Participants

Patients undergoing ambulatory hysteroscopy at Hainan General Hospital from February 2024 to May 2024 were recruited. Adults aged 18–64 years with ASA physical status I or II were eligible for the study. Body mass index (BMI) was recorded for all participants. All participants provided informed consent. Exclusion criteria included: severe intrauterine adhesions; respiratory or cardiovascular conditions; liver or kidney dysfunction; allergies or contraindications to the study drugs; recent use of sedatives or analgesics; psychiatric disorders; and refusal to cooperate with the study. Additionally, patients requiring conversion to tracheal intubation, those with surgeries lasting over 30 minutes, or cases where the surgical approach was altered intraoperatively were excluded.

### Intervention protocol

Upon entering the operating room, an intravenous line was established in the right forearm, and continuous monitoring was conducted for heart rate (HR), peripheral oxygen saturation (SpO_2_), electrocardiogram (ECG), bispectral index (BIS), and non-invasive blood pressure (NIBP). All patients underwent a standardized anesthesia protocol. Oxygen was continuously supplied through a face mask at 2 L/min. Ten minutes before the start of surgery, 50 mg of flurbiprofen axetil—an injectable non-steroidal anti-inflammatory drug and a prodrug of flurbiprofen—was administered intravenously over approximately 1 min. An initial intravenous dose of remimazolam was 0.2 mg/kg, succeeded by a continuous infusion at 1 mg$\cdot$kg^−1^$\cdot$h^−1^. Subsequently, esketamine was injected according to the assigned dose.

Surgical procedures commenced when the modified Observer’s Assessment of Alertness/Sedation (MOAA/S) score indicated a value of 0 (see Supplementary Table S2 for the MOAA/S scale). Upon completion of surgery, the remimazolam infusion was discontinued, followed by intravenous administration of 0.25 mg palonosetron. Subsequently, patients were transferred to the Post-Anesthesia Care Unit (PACU) for continuous monitoring of HR, NIBP, and SpO_2_. After completion of surgery, MOAA/S was assessed until patients responded readily to verbal calling (MOAA/S = 5), and anesthesia recovery time was recorded as the interval from the end of surgery to achieving MOAA/S = 5. After surgery, VAS pain scores were assessed at PACU admission (recovery) and at 4 h postoperatively. Rescue analgesia was administered if the PACU admission VAS exceeded 3, as a single intravenous dose of flurbiprofen axetil 50 mg. Adverse events (e.g., postoperative nausea and vomiting, coughing, and hiccups) were recorded during surgery and up to 24 h postoperatively.

Adverse events were managed according to a predefined rescue protocol. Respiratory depression (SpO_2_ < 95% for more than 30 s) was treated by increasing oxygen flow and performing airway maneuvers; assisted ventilation was provided when necessary. Hypotension (MAP decrease > 20% from baseline for more than 5 min) was treated with intravenous metaraminol 0.5 mg, which could be repeated as needed. Hypertension (MAP increase > 20% from baseline for more than 5 min) was managed with an additional bolus of remimazolam 0.1 mg/kg administered only after the intraoperative body-movement response (presence/absence of movement), which was used for the primary endpoint, had been observed and recorded. For bradycardia (HR < 60 beats/min sustained for more than 5 min), the surgical procedure was paused and atropine 0.5 mg was administered intravenously if needed. Postoperative nausea and vomiting occurring within 24 h were treated with intravenous dexamethasone 8 mg as rescue medication. Coughing, hiccups, and dizziness were managed conservatively with observation and vital-sign monitoring without pharmacologic intervention. Injection site pain was managed by slowing the injection rate.

Esketamine was administered at a starting dose of 0.3 mg/kg, with subsequent dose adjustments determined by patient responses using a biased-coin design (BCD) protocol. When the preceding patient showed body movement (positive response), the subsequent dose was increased by 0.03 mg/kg. Conversely, in the absence of movement (negative response), the subsequent dose was either reduced by 0.03 mg/kg or maintained at the same level, contingent upon a computer-generated random assignment. A nurse anesthetist, independent of the clinical team, prepared the esketamine and did not participate in subsequent clinical management or outcome assessment. Anesthesia providers, surgeons, intraoperative care providers, and patients were all blinded to the administered esketamine dose.

### Outcome measures

The primary outcome was defined as the ED_90_ of esketamine for suppressing intraoperative body movement. Secondary outcomes included: (1) anesthesia recovery time, defined as the time from the end of surgery to responsiveness to verbal calling (MOAA/S = 5); (2) incidence of postoperative nausea and vomiting (PONV) within 24 h postoperatively; (3) VAS pain scores at PACU admission (recovery) and 4 h postoperatively; and (4) adverse events occurring during surgery and within 24 h postoperatively, including respiratory depression (SpO_2_ < 95% for more than 30 s), hypotension (MAP decrease > 20% from baseline for more than 5 min), hypertension (MAP increase > 20% from baseline for more than 5 min), bradycardia (HR < 60 beats/min sustained for more than 5 min), injection site pain, dizziness, hiccups, and coughing.

### Statistical analysis

A minimum of 20 to 40 patients is necessary to achieve a stable estimation of the target dose using the biased-coin design under practical conditions [16]. This study included a total of 50 patients. The BCD method was used to assign doses to subsequent patients. In the determination of the target ED_90_ using the BCD method, the subsequent formula was employed: Probability (B) = (1 - $\tau$) / $\tau$ = (1 - 0.9) / 0.9, where B represents the target percentage. According to previous studies, body movement or frowning within 5 minutes of surgery starting was considered a positive response. If a patient’s response was negative, the subsequent patient was administered the same dose (with a probability of 89%) or a reduced dose (with a probability of 11%). This was randomly assigned by a computer. The ED_90_ was estimated using isotonic regression, and the corresponding confidence intervals (CI) were calculated using the isotonic regression package, specifically employing the "dosefind" and "quickinverse" functions. Continuous variables are reported as means ± standard deviation (SD) if normally distributed, otherwise as medians with interquartile ranges (IQR). Categorical data were summarized with frequencies and proportions.

## Results

**Fig. 1:**
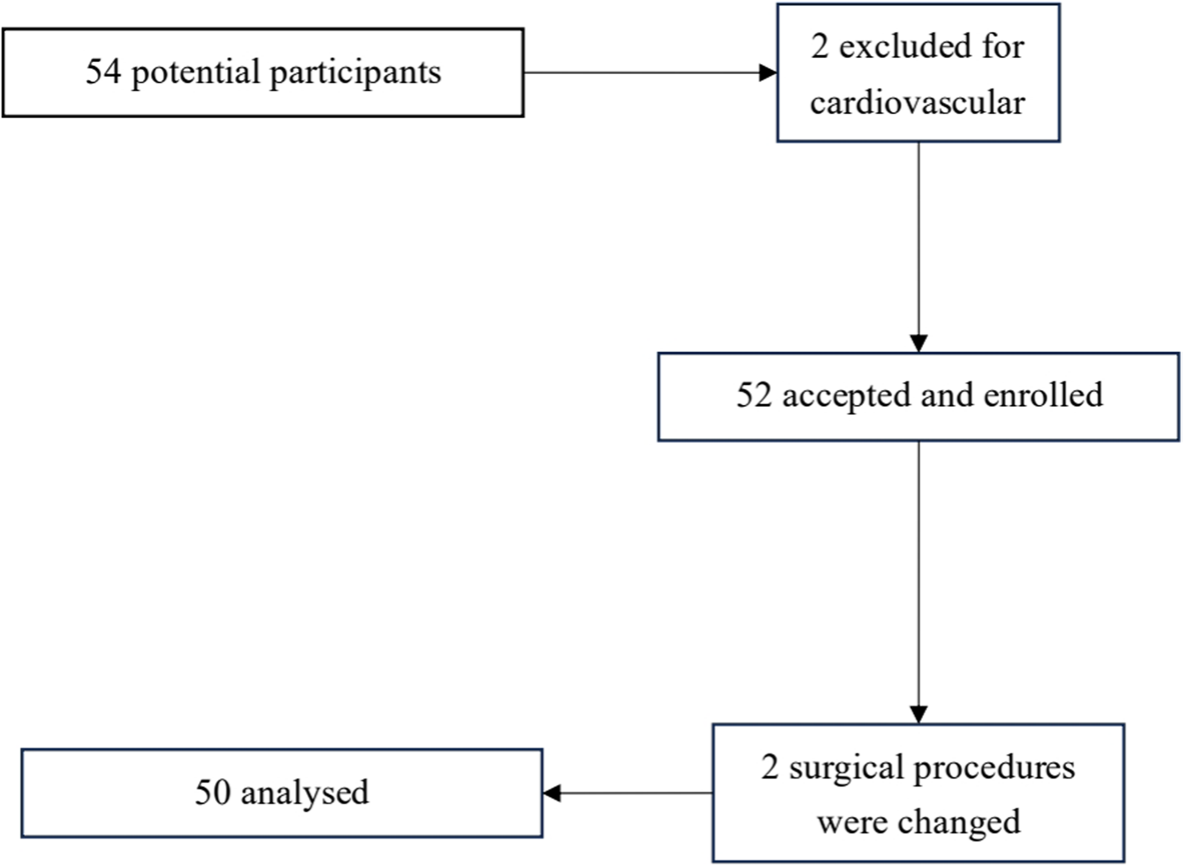
Patient enrollment flowchart showing screening process and exclusion criteria

Between February 2024 and May 2024, 54 patients were initially assessed for eligibility. Among these, 2 patients with a history of hypertension and 2 whose surgical procedures were modified (due to severe intrauterine adhesions requiring conversion to tracheal intubation and general anesthesia) were excluded. Consequently, fifty patients were recruited and completed the study (Fig. 1). Detailed baseline data for the enrolled population are provided in Table 1.

**Table 1:** Baseline characteristics of study participants (*n* = 50)

Characteristic	Value
Age (years)	41 ± 9.7
BMI (kg/m^2^)	22.6 ± 2.6
Weight (kg)	56.3 ± 7.4
Height (cm)	158.0 ± 4.2
ASA status (I/II)	16/34
Baseline HR (bpm)	73.6 ± 12.2
Baseline MAP (mmHg)	95.5 ± 10.9
SpO_2_ (%)	97.9 ± 1.4

Data presented as mean ± SD or n/N (%)

ASA  American Society of Anesthesiologists physical status classification

**Fig. 2:**
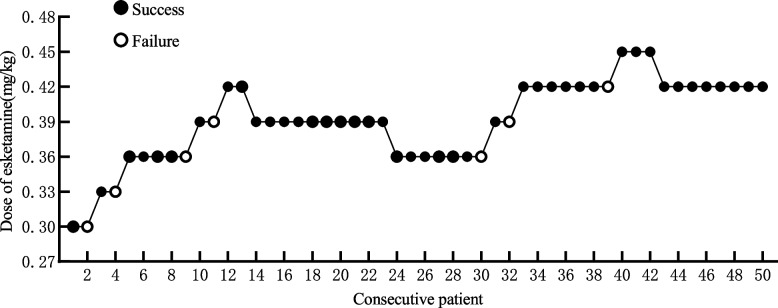
Biased-coin design (BCD) dose assignment algorithm with response-dependent adjustments

Esketamine doses ranged from 0.30 mg/kg to 0.45 mg/kg, with dose assignment guided by biased-coin design (BCD) as shown in Fig. 2. Of the participants, 2 received 0.30 mg/kg of esketamine with 1 exhibiting a negative body movement response; 2 received 0.33 mg/kg with 1 showing a negative response; 12 received 0.36 mg/kg with 10 demonstrating a negative response; 14 received 0.39 mg/kg with 12 showing a negative response; 17 received 0.42 mg/kg with 16 demonstrating a negative response; and 3 received 0.45 mg/kg with all showing a negative response. Using isotonic regression, the ED_90_ of esketamine for inhibiting intraoperative motor responses was estimated at 0.405 mg/kg (95% CI: 0.370–0.494).

Secondary outcomes showed that the mean anesthesia recovery time (from the end of surgery to MOAA/S = 5) was 12.1±2.0 min. Hemodynamic variables (HR and MAP) measured at five predefined timepoints remained stable throughout the perioperative period; descriptive data for each timepoint are shown in Supplementary Table S1. No cases of respiratory depression, hypertension, hypotension, or bradycardia were observed. Mild dizziness occurred in 19 patients (38%) within the first two hours postoperatively. Nausea, coughing, and hiccups were each reported in 2 patients (4%), while 1 patient (2%) experienced transient diplopia that resolved within four hours. Postoperative pain assessed using the VAS ranged from 0 to 4 at PACU admission (recovery), with two patients (4%) scoring 4; these patients met the predefined criterion for rescue analgesia (VAS > 3) and received a single intravenous dose of flurbiprofen axetil 50 mg. VAS scores decreased to 0–1 within four hours postoperatively. Clinical outcomes and adverse events are summarized in Table 2.

**Table 2:** Clinical outcomes and adverse events

Outcome	Value
ED_90_ (mg/kg)	0.405 (0.370–0.494)
Anesthesia recovery time (min)	12.1 ± 2.0
Respiratory depression	0 (0%)
Hypotension	0 (0%)
Hypertension	0 (0%)
Bradycardia	0 (0%)
Dizziness	19 (38%)
PONV	2 (4%)
Hiccups	2 (4%)
Coughing	2 (4%)
Diplopia	1 (2%)
VAS (recovery)	0–4
VAS (4 hours)	0–1

Data presented as mean ± SD, n (%), or range

*ED*_*90*_ 90% effective dose, *PONV* postoperative nausea and vomiting, *VAS* visual analogue scale

## Discussion

This study established that the ED_90_ of esketamine, in combination with remimazolam and preventive analgesia using flurbiprofen axetil, for suppressing intraoperative motor responses during ambulatory hysteroscopic surgery, was found to be 0.405 mg/kg (95% CI: 0.370–0.494). This result highlights a promising opioid-free anesthesia (OFA) protocol for ambulatory hysteroscopic surgery.

Ambulatory hysteroscopy offers benefits such as minimal trauma, rapid recovery, and high diagnostic accuracy, making it a preferred approach for managing intrauterine and cervical diseases [17]. To support the need for swift postoperative recovery, anesthetic protocols must minimize adverse effects and ensure rapid reversibility. To align with these needs, opioid-sparing or opioid-free anesthesia, as implemented in this study, has attracted significant attention since its introduction in the 1990s [18, 19]. OFA offers potential benefits, particularly in weight loss and cancer surgeries [20, 21], and its application has expanded to various procedures, including orthopedic, gynecological, head and neck, and thoracoscopic surgeries [22–25]. Commonly employed strategies in OFA integrates non-steroidal anti-inflammatory drugs (NSAIDs), local and regional analgesia, intravenous lidocaine, ketamine, dexmedetomidine, magnesium, and beta-blockers, providing a multimodal approach to effective analgesia while mitigating opioid-related risks [26]. Esketamine, known for its sedative and analgesic effects, acts as a non-competitive NMDA receptor antagonist and is widely used in clinical practice [27, 28]. Intravenous esketamine acts within 30 seconds and has a duration of 30 40 minutes [29], making it suitable for short procedures such as hysteroscopy. However, its psychiatric side effects, including dissociation and nightmares, limit its standalone use. Combining esketamine with benzodiazepines like remimazolam mitigates these effects through GABA_A_ receptor-mediated central inhibition, enhancing patient comfort and safety [30].

Subclinical doses of esketamine (less than 0.5 mg/kg) are safe and effective for non-intubated general anesthesia [31]. The ED_90_ for esketamine in adult daytime painless hysteroscopy is 0.405 mg/kg, representing a 29.4% reduction compared to our earlier studies without flurbiprofen axetil (0.574 mg/kg) [32]. This suggests that flurbiprofen axetil can effectively reduce the amount of esketamine needed during daytime hysteroscopic procedures. With a single intravenous push, remimazolam takes effect within 1-3 minutes, causes minimal respiratory and circulatory depression [14], and is associated with a lower incidence of hypotension than propofol during anesthesia induction [33].

Pain during hysteroscopic surgery is a multifactorial phenomenon involving various mechanisms [34]. The initial source of pain arises from cervical manipulation, which includes procedures such as grasping, cannulation, and dilation. These interventions stimulate visceral afferent fibers in the cervix and vagina, transmitting nociceptive signals via the pudendal and pelvic splanchnic nerves to the sacral spinal ganglia (S2- S4) [35]. This process is responsible for the sharp and localized pain that many patients experience during the procedure, especially during cervical dilation or introduction of the hysteroscope. In addition, uterine distension is another significant contributor to intraoperative pain during hysteroscopy. The process of expanding the uterine cavity to allow for clear visualization during the procedure can stimulate pain receptors within the uterus. Distension activates sympathetic fibers, with the resulting signals conveyed along the hypogastric pathways to the thoracolumbar spinal ganglia (T12-L2). This results in a deep, cramp-like pain that is often described as a feeling of pressure or fullness within the lower abdomen. Uterine distension, particularly when combined with endometrial injury, can exacerbate pain and is associated with increased release of inflammatory mediators, including prostaglandins. Prostaglandins are key players in the inflammatory response, and their release during hysteroscopy promotes uterine contractions, which can lead to both intraoperative discomfort and delayed postoperative pain due to continued uterine activity [36].

Endometrial damage, whether from biopsy or other surgical interventions, further compounds pain during hysteroscopy. The destruction of the endometrium during these procedures triggers additional inflammatory pathways, including the release of prostaglandins, which induce uterine contractions [34]. These contractions, although necessary for procedural purposes, increase the intensity of pain by perpetuating uterine ischemia and mechanical stress on the uterine wall. Moreover, the presence of prostaglandins and other inflammatory mediators postoperatively contributes to delayed pain, which can last for several hours after the procedure [36]. This inflammatory pain, often characterized by a dull or aching sensation, is typically managed through the use of NSAIDs or other analgesics. In this study, we demonstrated that effective pain management during and after ambulatory hysteroscopy was achieved through preemptive analgesia with flurbiprofen axetil, which not only provided adequate intraoperative anesthesia but also significantly reduced postoperative discomfort. The results highlight the importance of addressing both inflammatory and nociceptive components of pain in ambulatory hysteroscopic surgery.

All patients successfully completed the surgery without significant respiratory or cardiovascular complications. Postoperative recovery was rapid, with mild, self-limiting adverse effects. Dizziness, a common adverse effect of esketamine, occurred in 38% of patients in this study. Adverse events including dizziness were evaluated immediately after awakening, which may have contributed to the relatively high observed incidence. The symptoms were mild and transient, resolving spontaneously without medical intervention, consistent with the known profile of low-dose esketamine [37] . Nevertheless, this finding highlights the need for careful monitoring during the early recovery period and for considering dose optimization or supportive measures in future studies to mitigate this effect. Other minor adverse events, including visual disturbances (2%), coughing (4%), hiccups (4%), and nausea (4%), resolved spontaneously or could potentially be mitigated with preventive strategies such as slower drug administration or adjunct therapies. These findings underscore the effectiveness of the opioid-free anesthetic protocol in providing adequate sedation, analgesia, and rapid recovery for ambulatory hysteroscopic surgeries.

The use of BCD methodology for dose estimation is another notable strength of this study. Unlike traditional up-and-down designs, which involve sequential dose adjustments based solely on the previous response, BCD incorporates a probabilistic bias to select the next dose, ensuring a more balanced distribution of doses around the target level. This design helps to more accurately pinpoint the effective dose that inhibits motor responses, such as the ED_90_, by minimizing the potential for large dose steps or outliers that could skew the estimation. This methodology has gained substantial traction in recent years, particularly in clinical trials involving anesthetic agents and other pharmacological interventions. For example, BCD has been successfully employed in determining the optimal dose of anesthetic agents such as dexmedetomidine and 2-chloroprocaine [38, 39]. By employing the BCD approach, our study provides a more reliable estimate of the ED_90_ for esketamine, helping to refine anesthetic protocols and improve patient safety.

This study has certain limitations: firstly, this study excluded patients with complex conditions such as severe intrauterine adhesions, which limits the generalizability of results to such populations. Secondly, the protocol specified a fixed-dose regimen for remimazolam, with a predefined loading dose and maintenance infusion rate. Additional research is required to assess the influence of different remimazolam doses on the ED_90_ of esketamine. The study was limited to surgeries lasting $\le$30 minutes, and the generalizability of the findings to longer procedures warrants additional investigation.

## Conclusion

In conclusion, the ED_90_ of esketamine, in combination with remimazolam and preventive analgesia using flurbiprofen axetil, for suppressing intraoperative motor responses during ambulatory hysteroscopic surgery, was 0.405 mg/kg (95% CI: 0.370–0.494).

## Supplementary Information

Supplementary Table S1. Hemodynamic and monitoring variables at predefined timepoints.

Supplementary Table S2. Modified Observer’s Assessment of Alertness/Sedation (MOAA/S) score.

## Data Availability

Data is provided within the supplementary information files
