# Supplementary material for: The 90% effective dose (ED90) of esketamine for inhibiting responses to intraoperative motor stimulation during ambulatory hysteroscopy: a biased-coin up-and-down sequential allocation trial
Source: BMC Anesthesiol. 2026 Feb 14;26:183. doi: 10.1186/s12871-026-03687-1 (PMC13011717; doi:10.1186/s12871-026-03687-1)
Supplement: Supplementary file 1 — Supplementary Table S1. Hemodynamic and monitoring variables at predefined timepoints. [file 12871_2026_3687_MOESM1_ESM.pdf]

Supplementary Table S1. Hemodynamic and monitoring variables at predefined timepoints (n = 50)

Variable	T0	T1	T2	T3	T4
HR (beats/min)	74 ± 12	76 ± 11	76 ± 10	75 ± 10	74 ± 9
MAP (mmHg)	95.5 ± 10.9	88.7 ± 10.7	91.1 ± 12.1	96.6 ± 10.7	91.6 ± 8.6
SpO2 (%)	97.9 ± 1.4	99.6 ± 0.8	99.5 ± 0.7	99.4 ± 0.8	99.7 ± 0.6
BIS	96.1 ± 1.7	71.7 ± 4.0	74.1 ± 4.9	74.5 ± 4.9	85.2 ± 3.2

Data are presented as mean ± SD. T0: after entering the operating room and calming; T1: immediately after completion of esketamine injection; T2: at hysteroscope insertion; T3: 5 min after hysteroscope insertion; T4: at awakening. MAP, mean arterial pressure; SpO2, peripheral oxygen saturation; BIS, bispectral index.
