# Supplementary material for: The 90% effective dose (ED90) of esketamine for inhibiting responses to intraoperative motor stimulation during ambulatory hysteroscopy: a biased-coin up-and-down sequential allocation trial
Source: BMC Anesthesiol. 2026 Feb 14;26:183. doi: 10.1186/s12871-026-03687-1 (PMC13011717; doi:10.1186/s12871-026-03687-1)
Supplement: Supplementary file 2 — Supplementary Table S2. Modified Observer’s Assessment of Alertness/Sedation (MOAA/S) score. [file 12871_2026_3687_MOESM2_ESM.pdf]

Supplementary Table S2. Modified Observer's Assessment of Alertness/Sedation (MOAA/S) scale

Score	Response
5	Responds readily to name spoken in a normal tone.
4	Lethargic response to name spoken in a normal tone.
3	Responds only after name is called loudly and/or repeatedly.
2	Responds only after mild prodding or shaking (tactile stimulation).
1	Responds only after painful stimulation.
0	No response after painful stimulation.

MOAA/S: modified Observer's Assessment of Alertness/Sedation.
